# Suicide Prevention Among College Students Before and During the COVID-19 Pandemic: Protocol for a Systematic Review and Meta-analysis

**DOI:** 10.2196/26948

**Published:** 2021-05-17

**Authors:** Yunyu Xiao, Rachel Hinrichs, Nina Johnson, Amanda McKinley, Joan Carlson, Jon Agley, Paul Siu Fai Yip

**Affiliations:** 1 School of Social Work Indiana University–Purdue University Indianapolis Indianapolis, IN United States; 2 School of Social Work Indiana University–Bloomington Bloomington, IN United States; 3 IUPUI University Library Indiana University–Purdue University Indianapolis Indianapolis, IN United States; 4 School of Public Health Indiana University Bloomington Bloomington, IN United States; 5 Department of Social Work and Social Administration University of Hong Kong Hong Kong China (Hong Kong)

**Keywords:** suicide, suicidal prevention, college, university, health disparities, equity, suicidal ideation, suicide attempt, COVID-19, college student, young adult, disparity, review

## Abstract

**Background:**

Suicide is the second leading cause of death for college-aged individuals worldwide and in the United States. Recent studies have identified preliminary evidence of widening disparities in suicidal behaviors across sex, sexual orientation, race/ethnicity, age, and socioeconomic status among college students. Few systematic reviews and meta-analyses offer a comprehensive understanding of on-campus and off-campus suicide interventions, nor is collated information available for different types of screening, assessment, treatment, and postvention plans. Further challenges have been identified since the COVID-19 pandemic, calling for cost-effective and innovative interventions to address increased rates of suicidal behaviors among college students facing unprecedented stressors.

**Objective:**

This research protocol describes the first systematic review and meta-analysis to identify the most effective and cost-effective intervention components for universal and targeted (indicated and selected) suicide prevention among college students in a global context. Special attention will be placed on disparities in suicide prevention across sociodemographic subgroups, inclusive interventions beyond campus, global context, and intervention responses to the COVID-19 pandemic.

**Methods:**

A sensitive search strategy will be executed across MEDLINE (Ovid), EMBASE, PsycINFO (EBSCO), ERIC (EBSCO), Cochrane Library, Dissertations and Theses Global (ProQuest), Scopus, Global Index Medicus, SciELO, African Journals Online, Global Health (CABI), and Google Scholar. Data extraction and evaluation will be conducted by three independent researchers. Risk of bias will be assessed. A multilevel meta-regression model and subgroup analysis will be used to analyze the data and estimate effect sizes.

**Results:**

The initial search was completed in December 2020 and updated with additional other-language studies in March 2020. We expect the results to be submitted for publication in mid-2021.

**Conclusions:**

Despite increasing rates of suicidal behaviors among college students, few preventative efforts have targeted this population, and fewer focus on factors that might place specific demographic groups at heightened risk. The impact of COVID-19 on suicidal behaviors among college students highlights and exacerbates the urgent need for rapid and effective interventions that might differ from traditional approaches. This equity-focused study will address these gaps and provide a valuable analysis of the effectiveness of suicide prevention programs and interventions. Findings will inform clinicians, researchers, policy makers, families, and organizations about evidence-based interventions for reducing the gaps in the suicide crisis among college students from different sociodemographic groups.

**Trial Registration:**

PROSPERO CRD42020225429; https://www.crd.york.ac.uk/prospero/display_record.php?RecordID=225429

**International Registered Report Identifier (IRRID):**

DERR1-10.2196/26948

## Introduction

### Background

Suicide is the second leading cause of death for college-aged individuals worldwide and in the United States [1-7]. Globally, results from the World Health Organization World Mental Health International College Student Surveys indicated that 32.7% of college students seriously thought about suicide, and 4.3% attempted suicide, between 2014 and 2017 [3]. In the United States, one-fifth of college student participants in a recent national survey reported suicidal ideation, with 9% reporting suicide attempts [4]. Between 2007 and 2017, past-year suicidal ideation among college students nearly doubled (from 5.8% to 10.8%) [7]. Based on the Household Pulse Survey by the Centers for Disease Control and Prevention (CDC) conducted from February 17 to March 1, 2021, 42.2% of participants aged 18-29 years reported indicators of depression in the past week [8]. Notably, colleges and universities (hereafter “colleges”) have been identified as potential sites for suicide clusters where a substantial number of suicides could occur rapidly within a short time frame [9]. The trauma associated with exposure to a young person’s suicide significantly increases widespread anxiety and panic, and causes prolonged grief across victims, families, and communities [10]. There is an urgent need for research to develop effective, innovative, and accessible suicide prevention programs and interventions for college students.

In addition, recent studies have identified preliminary evidence of widening disparities in suicide across sex, sexual orientation, race/ethnicity, age, and socioeconomic status subgroups among college students [4,11,12]. Since 2000, female college students have reported a higher prevalence of suicidal ideation, planning, and attempts than their male counterparts in the United States [13,14]. Bisexual and transgender students were 2-3 times more likely to report suicidality than heterosexual and gay/lesbian students in 2015 [4]. In 2017, Black college students reported the highest rate of suicide attempts among college students (2.6% versus 1.4% among White students) [11]. There are sociodemographic differences in barriers to using mental health services on college campuses [12,15]. However, evidence-based suicide programs tailored to meet the unique needs of specific demographic groups are few. There is a need to develop culturally adaptive suicide interventions, given emerging evidence that experiences of structural discrimination, minority stress, adverse childhood experiences, social discord, and cultural sanctions might disproportionately affect the risk of suicidal behavior [16,17].

Further challenges for student mental health have been identified during the COVID-19 pandemic. In a recent CDC survey, young adults aged 18-24 years reported significantly greater rates of suicidal ideation than the general population during the pandemic (25.5% versus 10.7%) [18]. However, existing studies have focused on primary and secondary school students [19,20], and actions to tackle the impact of the COVID-19 pandemic on mental health among college students has not been comprehensively understood. The new challenge calls for proactive and effective responses from policy makers, researchers, and the global community to prevent youth suicide [21,22]. Telepsychiatry interventions and digital tools (eg, mobile apps, internet chatbots, videoconferencing) have proliferated rapidly in response to the COVID-19 emergency [23]. It is therefore important to review suicide prevention studies conducted after the onset of the COVID-19 pandemic to address pandemic-specific suicide risk [21]. If such studies have been published, preliminary results should also be synthesized, and service gaps identified [22].

### Rationale

Suicidal behaviors among college students can have wide-ranging adverse effects on well-being and development, including low academic achievement [24,25], chronic physical health conditions [26], and reduced labor market performance [27]. Early identification, effective treatment, and appropriate interventions for students have the potential to save students’ lives and improve societal well-being and social capital [3]. Despite recent attention to the alarming rates of suicidal behaviors among college students [3,4,7,28], there has been less research comparatively addressing suicide prevention and early intervention for college students than for primary and secondary school students [29]. This is troubling because the college years represent a critical and unique developmental stage [30] characterized by dynamic social role transitions, new living situations, and changing relationships [31]. It is important to understand and design college-specific intervention programs targeting the developmental stress-diathesis factors [32] during the transition from adolescence to emerging adulthood.

Existing systematic reviews on suicide prevention among college students are generally strong but are limited by their narrow foci in terms of populations, interventions, comparisons, and outcomes, as well as a lack of guidance from a theoretical framework. First, most previous reviews focus on symptomatic students [33], but evidence suggests a need for additional focus on those at risk but undiagnosed or untreated. To address this gap, this study will not be restricted to studies of students with a current diagnosis. Second, many campus counseling centers are underresourced, and college students have to use off-campus mental health services [12,34]. However, previous reviews predominantly focus on on-campus settings [35]. Conceptually, this may be related to a gap recently identified by the US Preventive Services Task Force: the lack of effective interventions linking clinical and community resources [36,37]. This study will extend the previous review by deliberately attempting to build a comprehensive understanding of available on-campus and off-campus services (eg, those in the community) and interventions. Third, consideration of the disparities faced by specific sociodemographic student groups is needed to improve screening and referral systems targeting high-risk groups [38]. Previous reviews exclude studies on interventions targeting high-risk populations (eg, sexual minorities), and no reviews have delineated differential intervention effects. This study will add to existing knowledge by exploring suicide interventions tailored to specific sociodemographic groups and assess their intervention outcomes.

Fourth, the interventions included in previous systematic reviews have been concentrated on gatekeeper programs with outcomes that do not directly measure suicidal behaviors (eg, many such programs assess secondary outcomes, including knowledge, skills, attitudes, or awareness) [33,35,39]. Our proposed study will include both primary suicide assessment (eg, suicidal ideation, plan, planning, and attempts) and secondary outcomes (eg, attitudes). Additionally, we plan to evaluate the cost-effectiveness (ie, costs of death prevented using the incremental cost-effectiveness ratio [ICER]) of the interventions where data are available, which has not been attempted in previous reviews.

Fifth, as one might expect, no reviews have examined the adaptability of suicide prevention programs in the context of the COVID-19 pandemic. This information will be important to inform the emerging transformation and proliferation of telepsychiatry in terms of the ways in which it might increase the accessibility of mental health services for college students [21-23,40]. Digital interventions provide the opportunity to reach at-risk college students who experience barriers to accessing traditional mental health services [41]. This study will add a specific focus on suicide interventions implemented during the COVID-19 pandemic when available.

**Figure 1:**
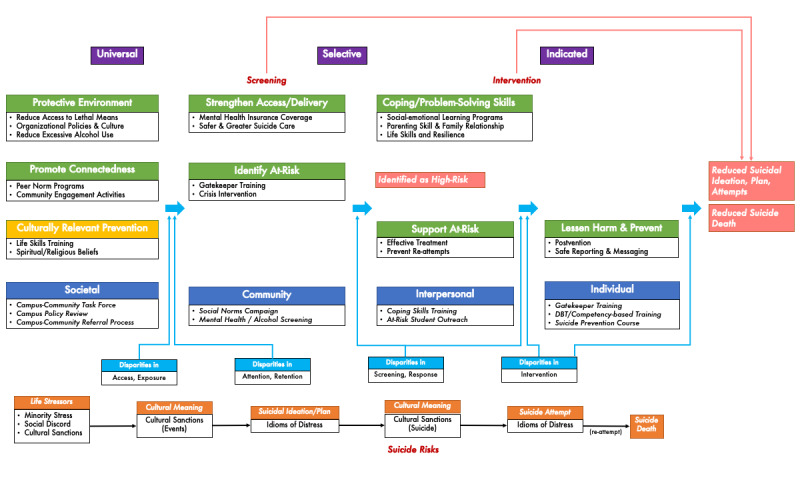
Logic model depicting potential sources of disparity in response to suicide prevention among college students.

Sixth, most existing reviews only consider studies conducted on college campuses in the United States [33,35,39], limiting the chance to learn from other developed and developing countries. This study will not limit the search criteria by geographic location, potentially adding informative global experiences to the existing body of knowledge. Finally, few reviews have adapted an evidence-based theoretical framework to guide the synthesis, with selected exceptions. Reviews that applied the two-paradigm framework (Clinical Intervention Zone, Prevention Zone) [35,39] and social-ecological model [42,43] suggest the need for more theoretically sound reviews with public health perspectives to offer a rigorous evaluation of existing efforts as a whole and within each level or paradigm. None of the existing systematic reviews have adopted a health equity framework [44,45] to guide the review process. This study will use a logic framework (Figure 1) based on PRISMA-E (Preferred Reporting Items for Systematic Review and Meta-Analysis – Equity; Multimedia Appendix 1), relevant guides, and previous empirical studies.

### Objectives

This protocol articulates our plan to conduct a comprehensive systematic review and meta-analysis to identify the most effective and cost-effective intervention components for universal and targeted (indicated and selected) suicide prevention among college students. This project will accomplish the following objectives: (1) analyze all student participants with a focus on disparities in suicide, (2) include on-campus and off-campus programs (in-person and virtual), (3) examine broader outcomes specific to suicidal behaviors, and other secondary outcomes that might reduce suicide (eg, knowledge, attitudes), (4) incorporate US and non-US studies, and (5) adhere to a theoretically developed logic framework (Figure 1). To offer a breadth of program evolution across various eras, our review will not limit the study time frame, though it will focus on the development of novel interventions prior to and during the COVID-19 pandemic. If a paucity of studies during the COVID-19 pandemic is identified, we will summarize the existing findings and reinforce the importance of understanding the potential effects of the COVID-19 pandemic on this body of literature [59].

We will attempt to clearly answer the following research questions:

1. What are the existing college-based and community-based suicide interventions for college students?
2. What are the common elements/types of suicide prevention interventions for college students?
3. What are the health and social outcomes of interest of the selected interventions?
4. Is there sufficient variability in interventions concerning the population, interventions, controls, and outcomes, based on the reported results and discussions?
5. Which components or combinations of components of suicide interventions are effective, and for which outcomes (primary versus secondary), demographic subgroups, settings (on-campus versus off-campus), and delivery method (in-person versus digital)?
6. Are there existing suicide intervention programs tailored to students from specific sociodemographic subpopulations? If so, what elements of the intervention are tailored?
7. Which suicide intervention has been the most efficacious and effective during the COVID-19 pandemic?
8. Which suicide intervention is the most cost-effective based on standard economic evaluation?

Cost-effectiveness will be measured by the values of ICERs that are available in the identified studies, or calculated given the availability of costs (eg, health care sector costs, nonmedical costs, and costs of productivity losses) reported in the studies [60].

Knowledge generated from our study will identify gaps in the evidence base and inform college leaders, policy actors, health care practitioners, clinicians, parents, and society about feasible approaches to screen and support at-risk college students across sociodemographic characteristics.

## Methods

### Overview

This protocol was developed using the 2015 PRISMA-P (Preferred Reporting Items for Systematic Review and Meta-Analysis Protocols) recommended checklist for systematic review protocols [61]. The systematic review and meta-analysis will be conducted and reported in accordance with the PRISMA statement [62] and the methods outlined in the Cochrane Handbook for Systematic Reviews of Interventions [63]. Given our specific focus on at-risk subpopulations (eg, racial/ethnic minorities, sexual minority students) and interventions aimed at reducing barriers to seeking help among college students across sex, race/ethnicity, sexual orientation, and socioeconomic status, we further adapted the PRISMA statement on equity-focused systematic reviews (PRISMA-E 2012) [46,64-67] to improve transparency and completeness in reporting health equity–focused systematic reviews, in addition to the previous PRISMA-E checklist developed by Moher and colleagues [68,69]. The review team is composed of researchers across disciplines with diverse backgrounds.

### Eligibility Criteria

#### Types of Participants

This review will consider studies involving college students (aged ≥18 years). We will also examine subpopulations across age, race/ethnicity, sex, sexual orientation, and socioeconomic status [70-73].

#### Types of Interventions

All programs that have at least one component intended to address suicide are eligible for inclusion. This includes programs that address general suicidal thoughts and behaviors, specific suicidal thoughts and behaviors, awareness of suicidal behaviors, help-seeking behaviors, or a combination of conditions. Included interventions will be broadly defined and include universal, indicated, or selected interventions at the individual, family, and school levels. Possible intervention mechanisms will include psychological (eg, cognitive behavioral therapy, psychodynamic psychotherapy), pharmacological (eg, antidepressants) [74], psychosocial (eg, restricting access to lethal means, screening for high-risk persons), educational (eg, education and awareness programs for the general public and professionals; media reporting of suicide), and physical (eg, exercise, occupational therapy) interventions to prevent/reduce suicidal ideation and behaviors. Interventions targeting secondary outcomes such as awareness of suicide and help-seeking behaviors will also be included. Interventions designed to primarily target behaviors that are risk factors for suicidal behaviors (eg, substance abuse) but that do not specifically address any of the components above will be excluded. Interventions focusing on gatekeepers (eg, families, teachers, health care providers) will be included. We will include randomized controlled trials (RCTs), pseudo-RCTs, observational pretest/posttest designs, and ecological or population-based studies that evaluate the effectiveness of suicide interventions.

#### Types of Prevention Settings

We will include all relevant settings, including campuses, community centers, digital tools, and hybrid (in-person and virtual) models. We will conceptualize digital tools, broadly, as internet-based interventions, chatbots, mobile device interventions, and social media interventions. We will not restrict inclusion criteria based on geographic location.

#### Types of Outcomes

The primary outcomes will include suicide-specific outcomes, suicidal ideation, suicidal thoughts, and suicidal behaviors (completed suicide or suicide attempts). If multiple measures of suicide are used, we will prioritize data extraction as follows: (1) validated questionnaires (eg, the Columbia-Suicide Severity Rating Scale [75] or Beck Scale for Suicide Ideation [76]), (2) clinician ratings, and (3) single-item analysis of other self-reported rating scales (eg, question 9 from the Patient Health Questionnaire-9 [77]). The secondary outcomes will include changes in suicide-related knowledge, attitudes, and behaviors. To examine equity-focused interventions, outcomes associated with inequality (eg, barriers to accessing care) will be included.

### Information Sources

We conducted a systematic search of the following databases from their inception until December 8, 2020: MEDLINE (Ovid), EMBASE, PsycINFO (EBSCO), ERIC (EBSCO), Cochrane Library, Dissertations and Theses Global (ProQuest), Scopus, and Google Scholar. For Google Scholar, all references on the first 10 pages, excluding books, will be retrieved. Including 10-20 pages (100-200 items) of references is suggested to achieve an optimal collection of the most relevant references [78]. On March 25, 2021, we expanded our search to include Global Index Medicus, SciELO, African Journals Online, and Global Health (CABI) in order to capture literature from low- and middle-income countries. Including such information sources may improve our ability to identify studies specifically relevant to suicide risks among sociodemographic subpopulations. Editorials, news items, conference proceedings and abstracts, patents, legal findings, and commentaries will be excluded. We will not restrict the search by language or publication date. We plan to use Google Translate (Google) for the purpose of data extraction of non–English language articles and to consult translators and colleagues proficient in the language, consistent with previous systematic reviews which included worldwide study context [79,80]. Researchers from our study team are native speakers of Chinese (YX) and proficient in Spanish (AM). We will screen relevant review articles and the reference lists of all included studies (backward search) for additional eligible studies. We will further screen studies that cited the included studies and relevant reviews (forward search). We will perform hand searches. We will include grey literature in ProQuest Dissertations and Theses dissemination from inception until December 8, 2020, in the systematic review, but not in the meta-analysis. We will also contact three experts in suicide prevention that we have identified to potentially obtain additional sources.

### Search Strategy

The database search strategies were developed by a health sciences librarian (RH) with expertise in literature searches. Known relevant articles collected by the authors were analyzed to select keywords and subject headings. An initial search strategy in MEDLINE Ovid was then iteratively developed by adding or removing additional keywords and subject headings until all known relevant articles were retrieved by the search, and no new relevant articles were found. The final search terms incorporated numerous headings, keywords, and publication types associated with three main concepts: college students, suicide, and intervention/prevention. In keeping with the health equity focus of the review, terms related to potentially underresourced college populations, such as nontraditional, commuter, foreign, international, or first-generation, were specifically included. Terms for prevention were purposely kept broad to encompass a wide range of possible interventions. The full search strategies for all information sources are provided in Multimedia Appendix 2.

### Study Records

#### Data Management

Identified articles are imported into EndNote 20 software (Clarivate Analytics), where duplicate references are removed. The remaining references are imported and managed in Covidence software (Veritas Health Innovation) for screening.

#### Selection Process

A total of two reviewers (NJ and AM) will independently screen the studies for eligibility (making a yes or no selection). Potential discrepancies during any step of the screening for inclusion/exclusion will be resolved by a third reviewer (YX). First, the reviewers will screen titles and abstracts identified in the databases. The team will then obtain and screen full-text articles. Studies that do not meet the eligibility criteria will be moved to an exclusion folder. All reviewers will strictly adhere to the inclusion and exclusion criteria. Final selected articles will be approved by the consensus of all reviewers and sent to an expert consultant for potential suggestions. The selection process will be displayed in a PRISMA flowchart [81].

### Logic Framework

Figure 1 illustrates the logic framework that we will employ during the review process. The logic framework recognizes that the causal chain of events linking preventative efforts to reduced suicidal behaviors can lead to differences in effects between socioeconomically disadvantaged and advantaged students in at least four ways: disparities in access/exposure, attention/retention, screening/response, and interventions.

### Data Collection Process

Data abstraction will occur independently and in duplicate using a piloted standard data collection form. Data extraction will include three categories: (1) study population and design, (2) intervention, and (3) outcome. Specific items in the extraction form will include study design, participant characteristics, geographic location, sample size, intervention methods, comparison intervention methods, primary and secondary outcomes, theoretical basis, mode of delivery, suicide prevention strategies, control condition, intensity and frequency of intervention, and treatment engagement (retention and attrition). Following PRISMA-E [44,45,67], we will include participant characteristics mapped to PROGRESS (place of residence, race/ethnicity/culture/language, occupation, gender/sex, religion, education, socioeconomic status, and social capital).

### Risk of Bias Assessment

For RCTs and pseudo-RCTs, reviewers will use the Cochrane Collaboration’s Risk of Bias tool [82]. Randomization procedures, bias, allocation, outcome assessor, reporting of findings, and losses to follow-up will be assessed. Studies will be classified as having a low, high, or unclear risk of bias. For non-RCTs (eg, controlled before/after designed studies), reviewers will use the Risk of Bias in Non-randomized Studies of Interventions (ROBINS-I) tool for evaluating the risk of bias in estimates [83]. The ROBINS-I assesses confounding participant selection, classification of the intervention, departures from the intended intervention, missing data, measurement of outcomes, selection of the reported results, and overall bias. Studies will be classified as being of low, moderate, serious, or critical risk of bias.

### Data Synthesis

#### Qualitative Synthesis

If the selected studies contain large amounts of heterogeneity or lack sufficient numbers to conduct the meta-analysis, we will follow the Narrative Synthesis in Systematic Reviews tool [84] and the PRISMA guidelines [81] to undertake a full narrative review. Following the PRISMA-E checklist [64], we will report both relative and absolute differences in intervention outcomes between sociodemographic groups. We will discuss the extent and limits of applicability to students across sex, race/ethnicity, age, and socioeconomic status. We will further provide implications for research, practice, or policy related to health equity in suicide prevention among college students where relevant (eg, types of interventions needed to address increasing suicide attempts among young Black males).

#### Meta-analysis

Should we identify a sufficient number of articles with low heterogeneity, we will conduct a meta-analysis among the final selected studies.

RevMan 5.3 will be used for all analyses. For continuous data, we will report the mean differences between groups and the 95% CI. We will calculate the standardized mean difference and 95% CI if different measurement tools were used for the same outcome, and the standard deviation if not reported [63]. We will use a random effects meta-analysis model given the possibility that there will be different types of interventions, heterogeneous characteristics of participants and comparators, and different intervention effects.

We will use *χ*^2^, I^2^, and T^2^ to assess heterogeneity [85]. Specifically, *χ*^2^ assesses the compatibility of observed differences in results (*χ*^2^ with *P*<.01 will be considered substantial heterogeneity). The I^2^ statistic represents the proportion of the total variation across studies due to heterogeneity (I^2^<40% indicates insignificant heterogeneity) [63], while T^2^ estimates the between-study variance in a random effects meta-analysis (T^2^>1 indicates substantial heterogeneity).

Sensitivity analysis will be conducted by examining whether excluding studies identified as having a greater risk of bias affects the effect sizes and comparisons between intervention and control groups.

Publication bias will be assessed by funnel plots and Egger test [86].

#### Sensitivity Analysis

With our comprehensive inclusion criteria, it is expected that the selected studies will include multiple study designs (eg, RCTs, non-RCTs, and observational studies). Recent studies report improved diagnostic accuracy after including different study designs in meta-analyses [86,87]. We plan to first conduct an analysis among combined RCTs and pseudo-RCTs, followed by separate subgroup analyses by study design to investigate the impact on the magnitude of the effect size observed for the included interventions.

#### Subgroup Analysis

Given the focus of this study on investigating health disparities, we plan to conduct subgroup analyses by sociodemographic characteristics and by pre-pandemic and pandemic periods when there are sufficient studies to do so. To increase the statistical rigor of our meta-analysis, we will include an independent meta-analysis statistician to review all our work as a blinded reviewer.

#### Evaluation of Cost-Effectiveness

We plan to evaluate the cost-effectiveness of the studies based on the reported ICER and the strength of evidence. We will classify interventions into cost-saving (better health outcomes and costs less than controlled group) or cost-neutral (ICER=0); very cost-effective ($0 < ICER ≤ $25,000 per quality-adjusted life-years [QALY] or life-years gained [LYG]); cost-effective ($25,000 < ICER ≤ $50,000 per QALY or LYG); marginally cost-effective ($50,000 < ICER ≤ $100,000 per QALY or LYG); or not cost-effective (>$100,000 per QALY or LYG) [60]. The strength of evidence (strong, supportive, or uncertain) will be assessed using criteria from a previous systematic review [88]. If there are no reported direct health care costs or evaluation of cost-effectiveness in the identified studies, we will summarize the data as reported in a previous review on depression intervention [89].

### Ethics and Dissemination

No ethical approval is required for this protocol and proposed systematic review as we will only use data from previously published papers that have themselves received ethics clearance and used proper informed consent procedures. The results of our systematic review and meta-analysis will be published in a peer-reviewed journal.

## Results

The systematic review and meta-analysis are currently in progress and expected to be finished by summer 2021. We welcome comments from reviewers and will be flexible in adjusting based on concerns related screening and data analysis to improve scientific rigor. Our final manuscript is expected to be submitted to peer-reviewed journals by August 2021.

## Discussion

### Principal Findings

Suicide is a significant public health crisis among college students worldwide [1-7]. However, there is a lack of research pertaining to effective suicide prevention programs among college students, particularly programs that could be tailored to target the unique needs of student subgroups (eg, sex, sexual orientation, race/ethnicity, age, and socioeconomic status). Although the impact of the COVID-19 pandemic on suicidal behaviors among college students has been recognized [18], little is known about possible suicide prevention programs for college students during the pandemic and their differences in crisis management that differ from pre–COVID-19 intervention programs.

Our systematic review and meta-analysis will address a significant lack of outcomes research examining the efficacy and effectiveness of available suicide prevention programs among college students. The strengths of our study are the inclusion of intervention and prevention programs with various study designs, settings, and modes of delivery across countries, and a specific focus on health equity. Our findings will inform clinicians, researchers, policy makers, families, and higher education organizations in reducing the gaps in the suicide crisis among college students from different sociodemographic subgroups.

### Limitations

Given the broad inclusion criteria, there may be high heterogeneity among the included studies. There may also be a small number of studies focused on newly developed interventions (eg, mobile technologies), which may have limited representativeness. We plan to follow established guidelines for handling heterogeneity [63,81,90,91]. We are minimizing the potential risk of studies being excluded during selection by following a rigorous protocol, conducting a prescreening training, including multiple coders, and employing cross-validation through a third reviewer. We will conduct sensitivity analysis by considering duplicate data extraction to minimize extraction errors [92,93]. We will include an external meta-analysis statistician to detect any scientific and statistical errors during the meta-analysis [94]. We are also aware that some community-based interventions may not have identified participants as college students, and thus, it may be difficult to identify data to examine any potential differences between on-campus and off-campus services. In such a case, we will summarize findings from the existing studies that report college students as the study sample. By submitting our protocol for review, we will also adjust for any critical threats not identified by the team prior to conducting the study.

### Implication

To the best of our knowledge, this will be the first systematic review and meta-analysis to examine the effectiveness of suicide prevention interventions among college students in such a wide-ranging and comprehensive manner. In addition, if possible, comparing pre-pandemic interventions and interventions during the pandemic could offer guidance for future initiatives and emerging needs.

## Appendix

Multimedia Appendix 1 Checklist of items for reporting equity-focused systematic reviews (PRISMA-E 2012).

Multimedia Appendix 2 Search strategies and updated number of results on March 24, 2021.

## Abbreviations

CDC: Centers for Disease Control and Prevention
ICER: incremental cost-effectiveness ratio
PRISMA: Preferred Reporting Items for Systematic Review and Meta-Analysis
RCT: randomized controlled trial
ROBINS-I: Risk of Bias in Non-randomized Studies of Interventions
