# Supplementary material for: Suicide Prevention Among College Students Before and During the COVID-19 Pandemic: Protocol for a Systematic Review and Meta-analysis
Source: JMIR Res Protoc. 2021 May 17;10(5):e26948. doi: 10.2196/26948 (PMC8130819; doi:10.2196/26948)
Supplement: Multimedia Appendix 2 [file resprot_v10i5e26948_app2.docx]

**Multimedia Appendix 2:** Search strategies and updated number of results on March 24, 2021

Search ran on 12/14/2020

## Medline Ovid

(((College* OR universit*) adj10 ((young adj (people OR adult*)) OR gatekeep* OR employee* OR enrolled OR student* OR hotspot*)) OR ((post-secondary OR tertiary) adj3 (school OR institution* OR education)) OR campus* OR graduate* OR undergraduate* OR (resident adj3 (advisor* OR assistant*)) OR (commuter adj3 school*) OR ((first generation OR international OR foreign OR nontraditional) adj3 student*) OR (young adult/ and universities/) OR ((nursing OR medical OR social work) adj (student* OR interns OR intern OR school)))

AND

(exp Suicide/ OR suicid* OR parasuicide)

AND

(intervention.tw or interventions.tw or prevent*.tw or program*.tw or policy.tw or policies.tw or ((mental.tw OR suicide.tw) adj3 (curriculum.tw OR awareness.tw OR screening.tw OR education.tw or training.tw or treatment.tw)) or counseling.tw or mass screening/ or help-seeking behavior/ or counseling/ OR hotline*.tw or phone*.tw. or telephone*.tw. OR Student Health Services/ OR exp Mental Health Services/)

not (letter OR editorial or news or comment).pt.

Results: 967

## EMBASE

((((college* OR universit*) NEAR/10 ('young people' OR 'young adults' OR 'young adult' OR gatekeep* OR employee* OR enrolled OR student* OR hotspot*)):ab,ti,kw) OR ((('post secondary' OR tertiary) NEAR/3 (school OR institution* OR education)):ab,ti,kw) OR campus*:ab,ti,kw OR graduate*:ab,ti,kw OR undergraduate*:ab,ti,kw OR ((resident NEAR/3 (advisor* OR assistant*)):ab,ti,kw) OR ((commuter NEAR/3 school*):ab,ti,kw) OR (((“first generation” OR international OR foreign OR nontraditional) NEAR/3 student*):ab,ti,kw) OR (((nursing OR medical OR 'social work') NEAR/1 (student* OR interns OR intern OR school)):ab,ti,kw) OR 'health student'/exp OR 'college student'/de OR 'graduate student'/de OR 'nontraditional student'/de OR 'phd student'/de OR 'postgraduate student'/de OR 'undergraduate student'/de OR 'university student'/de OR ((college/de OR 'university'/de) AND ('young adult'/de OR 'student'/de))) AND

('suicide'/exp OR 'suicidal behavior'/exp OR suicid*:ab,ti,kw OR parasuicide:ab,ti,kw)

AND

(intervention:ab,ti,kw OR interventions:ab,ti,kw OR prevent*:ab,ti,kw OR program*:ab,ti,kw OR policy:ab,ti,kw OR policies:ab,ti,kw OR (((mental OR suicide) NEAR/3 (curriculum OR awareness OR screening OR education OR training OR treatment)):ab,ti,kw) OR counseling:ab,ti,kw OR hotline*:ab,ti,kw OR phone*:ab,ti,kw OR telephone*:ab,ti,kw OR telemedicine:ab,ti,kw OR telehealth:ab,ti,kw OR telepsychiatry:ab,ti,kw OR 'mental health service'/exp OR 'telehealth'/exp OR 'mass screening'/de OR 'anonymous testing'/de OR 'help seeking behavior'/de OR 'counseling'/exp OR 'mass medium'/de OR 'social media'/de OR 'social media':ab,ti,kw OR 'mass media':ab,ti,kw OR (mobile NEAR/2 (technology OR app*)):ab,ti,kw)

NOT

([conference abstract]/lim OR [conference paper]/lim OR [conference review]/lim OR [data papers]/lim OR [editorial]/lim OR [erratum]/lim OR [letter]/lim OR [note]/lim)

Results: 1188

## PsycINFO

S1: (((College* OR universit*) N10 (“young people OR “young adult” OR “young adults” OR gatekeep* OR employee* OR enrolled OR student* OR hotspot*)) OR ((post-secondary OR tertiary) N3 (school OR institution* OR education)) OR campus* OR graduate* OR undergraduate* OR (resident N3 (advisor* OR assistant*)) OR (commuter N3 school*) OR ((first generation OR international OR foreign OR nontraditional) N3 student*) OR ((nursing OR medical OR social work) N1 (student* OR interns OR intern OR school)))

• Do in title field and abstract field under advanced search

S2: DE "College Students" OR DE "College Athletes" OR DE "Community College Students" OR DE "Education Students" OR DE "Junior College Students" OR DE "Nursing Students" OR DE "ROTC Students" OR DE "Graduate Students" OR DE "International Students" OR DE "Medical Students" OR DE "Postgraduate Students" OR DE "Transfer Students"

S3: suicid* OR parasuicid* OR DE "Suicide" OR DE "Attempted Suicide" OR DE "Suicidality"

S4:

DE "Suicide Prevention" OR DE "Screening" AND DE "Counseling" OR DE "Group Counseling" OR DE "Peer Counseling" OR DE "School Counseling" OR DE "Mental Health Services" OR DE "Community Mental Health Services" OR DE "Student Personnel Services" AND DE "Family Intervention" OR DE "Group Intervention" OR DE "School Based Intervention" OR DE "Preventive Mental Health Services" or DE "Help Seeking Behavior" OR DE "Telemedicine" OR DE "Online Therapy" OR DE "Teleconsultation" OR DE "Telepsychiatry" OR DE "Telepsychology" OR DE "Mass Media" OR DE "Social Media"

S5: intervention or interventions or prevent* or program* or policy or policies or ((mental OR suicide) N3 (curriculum OR awareness OR screening OR education or training or treatment)) or counseling OR hotline* or phone* or telephone* or “mass media” OR “social media” OR telemedicine OR telehealth OR telepsychiatry OR “mobile application” OR “mobile applications” OR “mobile app” OR “mobile technology”

• Do in title and abstract fields in advanced search

(S1 or S2) AND S3 AND (S4 or S5)

Limit to academic journals

Results: 1,188

## Scopus

TITLE-ABS((((College* OR universit*) W/10 ("young people" OR "young adults" OR "young adult" OR gatekeep* OR employee* OR enrolled OR student* OR hotspot*)) OR ((post-secondary OR tertiary) W/3 (school OR institution* OR education)) OR campus* OR graduate* OR undergraduate* OR (resident W/3 (advisor* OR assistant*)) OR (commuter W/3 school*) OR ((first generation OR international OR foreign OR nontraditional) W/3 student*) OR ((nursing OR medical OR "social work") W/1 (student* OR interns OR intern OR school))) AND (suicid* OR parasuicide) AND (intervention OR interventions OR prevent* OR program* OR policy OR policies OR ((mental OR suicide) W/3 (curriculum OR awareness OR screening OR education OR training OR treatment)) OR counseling OR "help-seeking behavior" OR hotline* OR phone* OR telephone* OR "student health services") )

*limit to articles and reviews only

Results: 1309 results

## ERIC

EBSCO

S1: (((College* OR universit*) N10 (“young people OR “young adult” OR “young adults” OR gatekeep* OR employee* OR enrolled OR student* OR hotspot*)) OR ((post-secondary OR tertiary) N3 (school OR institution* OR education)) OR campus* OR graduate* OR undergraduate* OR (resident N3 (advisor* OR assistant*)) OR (commuter N3 school*) OR ((first generation OR international OR foreign OR nontraditional) N3 student*) OR ((nursing OR medical OR social work) N1 (student* OR interns OR intern OR school)))

• Do in title field and abstract field under advanced search

S2: DE "College Students" OR DE "College Freshmen" OR DE "College Seniors" OR DE "College Transfer Students" OR DE "First Generation College Students" OR DE "Graduate Students" OR DE "In State Students" OR DE "On Campus Students" OR DE "Out of State Students" OR DE "Preservice Teachers" OR DE "Two Year College Students" OR DE "Undergraduate Students" OR DE "Law Students" OR DE "Medical Students"

S3: suicid* OR parasuicid* OR DE "Suicide"

S4: DE "Health Education" OR DE "Screening Tests" AND DE "Counseling" OR DE "Family Counseling" OR DE "Group Counseling" OR DE "Individual Counseling" OR DE "Peer Counseling" OR DE "Help Seeking" OR DE "Student Personnel Services" OR DE "Crisis Intervention"

S5: intervention or interventions or prevent* or program* or policy or policies or ((mental OR suicide) N3 (curriculum OR awareness OR screening OR education or training or treatment)) or counseling OR hotline* or phone* or telephone* OR telemedicine OR telehealth OR telepsychiatry OR “mass media” OR “social media” OR “mobile application” OR “mobile applications” OR “mobile app” OR “Mobile technology”

Do in title and abstract fields in advanced search

(S1 or S2) AND S3 AND (S4 or S5)

Excluded books

Results: 448 (should probably exclude books)

## Cochrane Library

All searched in title, abstract, keyword fields:

(((university OR college) NEAR/3 (employee* OR student OR students OR enrolled)) OR ((post-secondary OR tertiary) NEAR/3 (school OR institution* OR education)) OR campus* OR graduate* OR undergraduate* OR (resident NEAR/3 (advisor* OR assistant*)) OR (commuter NEAR/3 school*) OR ((first generation OR international OR foreign OR nontraditional) NEAR/3 student*) OR ((nursing OR medical OR social work) NEXT (student* OR interns OR intern OR school)))

AND

(Suicid* OR parasuicid*)

AND

(intervention or interventions or prevent* or program* or policy or policies or ((mental OR suicide) NEAR/3 (curriculum OR awareness OR screening OR education or training or treatment)) or counseling OR hotline* or phone* or telephone* OR “help-seeking” OR “student personnel services” OR “student health services” OR telemedicine OR telehealth OR telepsychiatry OR “mass media” OR “social media” OR “mobile application” OR “mobile applications” OR “mobile app” OR “Mobile technology”)

Results: 146 trials

## Google Scholar

college|university student|employee|staff "suicide" intervention|program|prevention|policy|telemedicine|counseling|screening|education|awareness|mobile

Download 10 pages of results, excluding books and citation-only

Downloaded 97 records

## ProQuest Dissertations and Theses

ab,ti(((intervention OR interventions OR prevent* OR program* OR policy OR policies OR ((mental OR suicide) NEAR/3 (curriculum OR awareness OR screening OR education OR training OR treatment)) OR counseling OR hotline* OR phone* OR telephone* OR "help-seeking" OR "student personnel services" OR "student health services" OR telemedicine OR telehealth OR telepsychiatry OR “mass media” OR “social media” OR “mobile application” OR “mobile applications” OR “mobile app” OR “mobile technology”))) AND ab,ti(((Suicid* OR parasuicid*))) AND ab,ti(((post-secondary OR tertiary) NEAR/3 (school OR institution* OR education)) OR campus* OR graduate* OR undergraduate* OR (resident NEAR/3 (advisor* OR assistant*)) OR (commuter NEAR/3 school*) OR ((first-generation OR international OR foreign OR nontraditional) NEAR/3 student*) OR ((nursing OR medical OR "social work") NEAR/1 (student* OR interns OR intern OR school)))

No limits

Results: 276

## Global Index Medicus

Use advanced search. Title/Abstract/Subject

(((college* OR universit*) AND (employee* OR staff OR student* OR first-generation OR international OR foreign OR nontraditional)) OR "resident advisors" OR "resident assistants" OR "resident advisor" OR "resident assistant" OR "medical students" OR "nursing students" OR "Social work students")

AND

(Suicid* OR parasuicid*)

Results: 248

Search ran and added on 3/24/2021

## Scielo

((((((college* OR universit*) AND (employee* OR staff OR student* OR first-generation OR international OR foreign OR nontraditional)) OR "resident advisors" OR "resident assistants" OR "resident advisor" OR "resident assistant" OR "medical students" OR "nursing students" OR "Social work students")) AND (Suicid* OR parasuicid*))) AND ((intervention or interventions or prevent* or program* or policy or policies or ((mental OR suicide) AND (curriculum OR awareness OR screening OR education or training or treatment)) or counseling OR hotline* or phone* or telephone* OR "help-seeking" OR "student personnel services" OR "student health services" OR telemedicine OR telehealth OR telepsychiatry OR "mass media" OR "social media" OR "mobile application" OR "mobile applications" OR "mobile app" OR "Mobile technology"))

Results: 82

## African Journals Online

Uses a “Google-powered” search

college|university student|employee|staff "suicide" intervention|program|prevention|policy|telemedicine|counseling|screening|education|awareness|mobile

Download 5 pages of results

42 articles downloaded

**Global Health (CABI)**

(((university OR college) NEAR/3 (employee* OR student OR students OR enrolled)) OR ((post-secondary OR tertiary) NEAR/3 (school OR institution* OR education)) OR campus* OR graduate* OR undergraduate* OR (resident NEAR/3 (advisor* OR assistant*)) OR (commuter NEAR/3 school*) OR ((first generation OR international OR foreign OR nontraditional) NEAR/3 student*) OR ((nursing OR medical OR social work) NEAR/1 (student* OR interns OR intern OR school)))

AND

(Suicid* OR parasuicid*)

AND

(intervention or interventions or prevent* or program* or policy or policies or ((mental OR suicide) NEAR/3 (curriculum OR awareness OR screening OR education or training or treatment)) or counseling OR hotline* or phone* or telephone* OR “help-seeking” OR “student personnel services” OR “student health services” OR telemedicine OR telehealth OR telepsychiatry OR “mass media” OR “social media” OR “mobile application” OR “mobile applications” OR “mobile app” OR “Mobile technology”)

Results: 384
